# Supplementary material for: qPCR in a suitcase for rapid Plasmodium falciparum and Plasmodium vivax surveillance in Ethiopia
Source: PLOS Glob Public Health. 2022 Jul 27;2(7):e0000454. doi: 10.1371/journal.pgph.0000454 (PMC10021179; doi:10.1371/journal.pgph.0000454)
Supplement: S1 Protocols — (DOCX) [file pgph.0000454.s001.docx]

**qPCR in a suitcase for rapid *Plasmodium falciparum* and *Plasmodium vivax* surveillance in Ethiopia**

Lise Carlier, Sarah Cate Baker, Tiffany Huwe, Delenasaw Yewhalaw, Werissaw Haileselassie, Cristian Koepfli

**Supplementary file S1: Laboratory Protocols**

**A) DNA extraction**

Reference: Tenfold difference in DNA recovery rate: systematic comparison of whole blood vs. dried blood spot sample collection for malaria molecular surveillance

Holzschuh A and Koepfli C

Malaria Journal 2022

This protocol is for use with the NucleoMag blood 200 µL protocol (Macherey-Nagel catalogue no. 744501.4). As DNA is extracted from 100 µL blood, reagent volumes are adjusted. All steps are done at room temperature. All mixing steps are done at 1200 RPM. Use a reagents reservoir and 12-channel pipettes to dispense buffers. When dispensing reagents, avoid touching the rim of the wells and use the same 12 tips for whole plate.

1. Aliquot 100 µL blood per sample in Square-Well Block (S-Block) (use 1 rack of 300 µL filter tips)
2. Per sample: Mix 10 µL Proteinase K with 40 µL MBL1
   1. For 1 plate: 1 mL Proteinase K, 4 mL MBL1
   2. Add 50 µL per sample
   3. Mix at 1200 rpm for 10 minutes
3. Per sample: Mix 12.5 µL beads + 50 µL MBL 2
   1. Before use, shake beads very well for 1 minute
   2. For 1 plate: 1250 µL beads, 5 mL MBL 2
   3. Dispense 62.5 µL beads/MBS2. Beads settle very quickly. Mix by petting up and down each time before taking up to ensure even distribution of the beads across the plate.

1. Add 100 µL MBL2
   1. Mix on shaker for 5 minutes
   2. Put plate on magnet for 2 minutes
   3. Set pipette to 300 µL and remove and discard supernatant. Use new filter tips for each sample (use 1 rack of 300 µL filter tips). As the total volume is >300 µL, remove the supernatant in 2 steps.
2. Add 500 µL MBL3 (1^st^ wash) (As the total volume is >300 µL, add buffer in 2 steps)
   1. Mix on shaker for 5 minutes
   2. Put plate on magnet for 2 minutes.
   3. Remove and discard supernatant. Use new filter tips for each sample (use 1 rack of 300 µL filter tips). As the total volume is >300 µL, remove the supernatant in 2 steps.
3. Add 300 µL MBL3 (2^nd^ wash)
   1. Mix on shaker for 5 minutes.
   2. Put plate on magnet for 2 minutes.

Remove and discard supernatant (use 1 rack of 300 µL filter tips)

1. Add 300 µL 80% Ethanol
   1. Mix on shaker for 5 minutes.
   2. Put plate on magnet for 2 minutes.

Remove and discard supernatant (non-filter tips can be used)

- 1. Air-dry for 15 minutes (also empty + dry reservoir)

1. Add 100 µL MBL5 (elution buffer)
   1. Mix on shaker for 5 minutes.
   2. Put plate on magnet for 2 minutes.
   3. Transfer DNA to storage plate (use 1 rack of 300 µL filter tips)

**B) *Plasmodium falciparum* *var*ATS qPCR**

Reference: Ultra-sensitive detection of *Plasmodium falciparum* by amplification of multi-copy subtelomeric targets.

Hofmann N, Mwingira F, Shekalaghe S, Robinson LJ, Mueller I, Felger I

PLoS Medicine 2015

Prepare primers and probe to **10 µM**

qPCR master mix used: QuantaBio PerfeCTa qPCR ToughMix (catalogue no. 95112-012). Alternatively, the ThermoFisher TaqMan FastAdvanced Master Mix (catalogue no. 4444556) can be used.

Reaction mix setup

varATS forward+reverse primers 0.48 µL

varATS_probe 0.48 µL

PerfeCTa Though Mix 6 µL

H_2_O 1.04 µL

DNA 4 µL

Total 12 µL

Cycling conditions

50° 2 min

95° 2 min

95° 10 sec

55° 30 sec 45 cycles

Primer and probe sequences

Pf_varATS forward CCCATACACAACCAAYTGGA

Pf_varATS reverse TTCGCACATATCTCTATGTCTATCT

Pf_varATS probe 6-FAM-TRTTCCATAAATGGT-NFQ-MGB

**C) *Plasmodium vivax* *cox1* qPCR**

Reference: *Plasmodium vivax* molecular diagnostics in community surveys: pitfalls and solutions.

Gruenberg M, Moniz CA, Hofmann NE, Wampfler R, Koepfli C, Mueller I, Monteiro WM, Lacerda M, de Melo GC, Kuehn A, Siqueira AM, Felger I

Malaria Journal 2018

Prepare primers and probe to **10 µM**

qPCR master mix used: QuantaBio PerfeCTa qPCR ToughMix (catalogue no. 95112-012). Alternatively, the ThermoFisher TaqMan FastAdvanced Master Mix (catalogue no. 4444556) can be used.

Reaction mix setup

cox1 forward+reverse primers 0.48 µL

cox1 probe 0.48 µL

FastAdvanced MM 6 µL

H_2_O 1.04 µL

DNA 4 µL

Total 12 µL

Cycling conditions

50° 2 min

95° 2 min

95° 10 sec

60° 30 sec 45 cycles

Primer and probe sequences

Pv_cox1 forward TTATATCCACCATTAAGTACATCACTT

Pv_cox1 reverse AACCTTTAGATCTTAGATGCATTACA

Pv_cox1 probe VIC-CCTGTTGCAGTAGATGTTATCATTG-BHQ1
