# Supplementary material for: qPCR in a suitcase for rapid Plasmodium falciparum and Plasmodium vivax surveillance in Ethiopia
Source: PLOS Glob Public Health. 2022 Jul 27;2(7):e0000454. doi: 10.1371/journal.pgph.0000454 (PMC10021179; doi:10.1371/journal.pgph.0000454)
Supplement: Attachment — Submitted filename: Ziway_Response-to-Reviewers.docx [file pgph.0000454.s004.docx]

We thank the reviewers for their detailed comments and suggestions for improvement. We are confident we were able to address all comments.

Reviewer comments are in black.

Our responses are in blue.

Reviewer #1: The author here tried to demonstrate the adaptability of a portable suitcase-based laboratory facility, which is able to detect the occurrence of genetic material of Plasmodium falciparum and P. vivax at a makeshift laboratory in Ethiopia. This portable facility is projected as a rapid tool for sensitive surveillance of malaria parasite infection and may be adapted in malaria control activities. However, the author, in addition to demonstration of the portable lab facility and its comparability with that of traditional diagnosis methods like microscopy and RDT; they could have validated the sensitivity and specificity of qPCR assay with some controls at the field site. Below are few concerns which should be clarified before the article get published.

Minor Revision
Line 150 typing error in 12-channel pipettes volume mentioned 1-20ul instead of 1-10ul as seen in fig.1

Answer: We have corrected this typo.

Line 150 typing error in volume mentioned 20-300ul instead of 30-300ul as seen in fig.1

Answer: We have corrected this typo.

Line 208 may clarify the “high quality data”

Answer: We have clarified this sentence as follows (lines 230-231):

“[…] high quality data on *P. falciparum* and *P. vivax* infection status with a limit of detection of <1 parasite µL blood […]”

Line 209 may change from “a period of few week” to precise 2-months which is mentioned in intro section earlier.
Table.1 should mention the waste-bin and microtube racks.

Answer: We did not carry waste bins to the field site (we bought plastic buckets locally).

We have added this to the results (line 166):

“Plastic buckets were purchased as waste bins.”

We have included one tube rack in Table 1. In case the reviewer refers to qPCR tube racks, those are not required as the MIC qPCR tubes come prepacked in racks. We have clarified this in table 1.

Table.1 should mention the steps at which filter tips were used.

Answer: We have clarified this in the protocol in the supplementary file. This information would be difficult to integrate into table 1, as no protocol details are given there.

Methods should mention the PCR condition so that the time assessment of the qPCR will be clear.

Answer: We have included detailed qPCR protcols in the supplementary File S1. This file is no titled “Supplementary File S1: Laboratory protocols”. We have also included this detail to the methods section (line 128):

“Detailed qPCR protocols are given in Supplementary File S1.”

Major Revision
1. Results described for P. falciparum screening was not matched with that of Venn diagram in fig.2 qPCR positives mention 51 instead of 52 as per fig.2; 13 positives from microscopy instead of 19 as per fig.2; 18 positives by RDT instead of 21 as per fig.2. qPCR positives in asymptomatic cases 4/1029 instead of 4/1021 as per fig.2.

Answer: We thank the reviewer for spotting these inaccuracies and have corrected them as follows:

- The total of qPCR positives is 52. This was a typo that we have corrected.

- We refer to the 13 samples that were microscopy and qPCR positive (not all 19 microscopy positive ones). We have clarified this sentence as follows (lines 184-185): “By local microscopy, only 13/52 qPCR positive samples were correctly diagnosed.”

- Likewise, we refer to the 18 samples that were positive by RDT and confirmed by microscopy. We have clarified this as follows (lines 186-187): “RDT was moderately more sensitive, with 18/52 qPCR positive samples detected by RDT.”

- The total number of asymptomatic samples was 1021. ‘1029’ was a typo that we have corrected.

2. Table.2 seems not that important and can be in supplementary files.

Answer: After consulting with all authors, we disagree with this assessment. Table 2 contains the detailed data of the qPCR screening. We believe this data should be kept as a table in the manuscript.

3. HRP-2/3 typing was not mentioned in the methods and seems to be performed at the central lab. The whole method of this typing should be mentioned and the results should be described in details.

Answer: The detailed protocol and validation is described in our publication that is referenced (our reference 34). We have added detail as follows (lines 137-139):

“In this assay, either *hrp2* or *hrp3* is multiplexed with a control gene, *serine-tRNA ligase*. Both targets are amplified with very high sensitivity and specificity, thus providing highly accurate data on deletion status [34].”

4. Why 4 sample with positive P. vivax results for microscopy and RDT were repeated for the qPCR and the same repetition is not done for 6 sample with positive *P. falciparum* results for microscopy and RDT.

Answer: We have clarified that repetition was also done for the *P. falciparum* samples (lines 188-189):

“They remained negative when the qPCR was repeated. Mix up at the health center during sample and data collection might have occurred.”

5. What is the “short turnaround time” in line 209, author should describe it.

Answer: We have clarified as follows (line 232):

“A short turnaround time of a few weeks […]”

This number derives from the fact that transmission from one human host takes approximately 6 weeks. The exact time frame differs among specific use cases and a detailed discussion would be beyond the scope of this manuscript.

6. Contradictory statement at different lines; line 213 mentioned need of pair of laboratory technicians enough to process for 2-plates/day, however line 217 mentioned molecular laboratory skills are needed to perform all these.

Answer: ‘laboratory technicians’ and ‘laboratory skills’ are different terms for the same, as laboratory technicians by definition need to have laboratory skills. We have added to following detail to clarify the amount of training required (lines 245-247):

“Based on training experiences in multiple malaria endemic countries, individuals with no prior laboratory experience can learn the skills to conduct DNA extractions and qPCR within two weeks (C. Koepfli, unpublished).”

7. The sensitivity and specificity are not experimented here in this research work. Even if the published primer and probes is adapted here, there should be an explanation of any effect on the whole assay due to reducing DNA extraction from 200ul to 100ul initial source of DNA. May be all of the positives and few negatives from the field sites should be rechecked at the central lab using the isolated DNA from field sites for showing accuracy and reproducibility of this qPCR assay.

Answer: We extensively validated the limit of detection in our previous publication (reference 28). We added the following details to our manuscript (line 126):

“Using the extraction method we chose for this study, the limit of detection of this assay is 0.3 parasites/µL blood [28].”

The specificity is only affected by possible cross-contamination. This depends primarily on the training and skills of the laboratory technicians. We have trained individuals who have extracted thousands of samples using the same protocol without any contamination issues (e.g. <https://pubmed.ncbi.nlm.nih.gov/33422001/> and <https://www.medrxiv.org/content/10.1101/2022.04.26.22274317v1>), but also seen contamination issues when people were not properly trained.

8. Why parasitaemia is not noted in this study?, as that may have helped in concluding the detection limit.

Answer: In order to obtain robust parasitemia estimates by qPCR, a large number of external standards need to be run along field samples. These standards could be derived either from high-density samples quantified my multiple expert microscopists, DNA quantified by digital PCR, or plasmids. None of these assays was feasible in our field setting, thus any absolute parasite density data by qPCR would not be reliable.

9. Why the whole assay of qPCR is singleplex, which seems to consume more time?

Answer: We used assays targeting multi-copy genes in P. falciparum and P. vivax that are highly sensitive. These assays have very different qPCR conditions (for example, the optimal annealing temperature for the Pf assay is 55°C, while it is 60°C for the Pv assay). Thus, they cannot be multiplexed. Multiplexed assays that were published have a much higher limit of detection and thus are not recommended to detect very low-density infections.

Also, the benefits of a multiplexed assay would be small. It only takes around 15 minutes to set up a qPCR plate, thus the savings in hands-on time would be small. The cost for one qPCR is only around USD 0.50 per sample.

10. Few discussions are needed for the insight to the successful demonstration of suitcase lab;
a) What are the chances of false positives at field site due to cross contamination? What is the measure to avoid it in this kind of setting like qPCR suitcase-lab?

Answer: We have added detail as follows (lines 254-257):

“The main risk of high-throughput manual DNA extraction in 96-well format is cross-contamination. This risk does not differ in a field laboratory compared to the same protocol being used in a reference laboratory, and can be minimized by proper training of personnel.”

b) Who performed all qPCR for n=1920 with what skill sets? and how could be those skills be adapted by the technicians should be discussed.

Answer: We have added the following detail (lines 245-247):

“Based on training experiences in multiple malaria endemic countries, individuals with no prior laboratory experience can learn the skills to conduct DNA extractions and qPCR within two weeks (C. Koepfli, unpublished).”

c) The n=1920 samples were successfully performed in 2 months. Here, through is presented as 2*96=192/day and according to that n=1920 should be completed in 10 days. So, it should be clearly mentioned the throughput because current throughput seems around 192/day DNA isolation and 48/day qPCR to complete n=1920 sample in 2-months.

Answer: We have clarified this as follows (lines 237-240):

“The two-month study period included laboratory setup (e.g. cleaning, and procurement of freezer), visits to health centers and communities before sampling began, and sample collection. The limiting factor in the present study was the number of individuals that could be sampled per day.”

We do not know why the reviewer concludes that only 48 samples per day can be run on the qPCR. The qPCR takes only approximately one hour, thus eight plates can be run on one day, resulting in 2x96 samples screened for Pf and Pv.

d) The limitation may be mentioned that could be the power cuts, which may affect whole reagent stored in -20°C deep freezer. Is there any alternative strategy for such situation? Addition of solar power backup for the related equipment may be an option to power cuts. Is it possible to share the data with central lab for interpret qPCR data as that can resolve the need of skilled personnel?

Answer: We did not test solar panels and thus cannot comment on the feasibility. As described in the discussion, we would recommend using a generator.

++++

Reviewer #2: Author describe the importance of molecular diagnosis using qPCR in a suitcase for rapid malaria surveillance in Ethiopia. The study is designed and conducted well with adequate sample size. However few question need to be answered before considering for publications.

1. the detail protocol of qPCR is missing.

Answer: We have included detailed qPCR protocols in the supplementary File S1.

2. author has provided the running cost but no mentioned about the equipment's/ suitcase cost.

Answer: We have added these details in the results section (lines 157-159):

“The cost for all instruments totals approximately USD16,900 (not including the laptop computer). Costs are as follows: qPCR instrument: USD13,000, plate shaker: USD300, magnetic block: USD700, 2 x multichannel pipette: $1000 per pipette, 3 x single channel pipette: USD300 per pipette.”

3. Author need to mentioned the level of technical expertise required to perform such test.

Answer: We have added the following detail (lines 245-247):

“Based on training experiences in multiple malaria endemic countries, individuals with no prior laboratory experience can learn the skills to conduct DNA extractions and qPCR within two weeks (C. Koepfli, unpublished).”

4. six individual were positive for P. falciparum by microscopy but fail to detect by qPCR, please justify

Answer: As explained above in response to reviewer 1, we had clarified that we repeated the qPCR. We agree that this number is higher than expected, but cannot provide any further explanation.

5. similarly 4 cases were positive for P. vivax by microscopy but fail to detect by qPCR, please justify

Answer: As for *P. falciparum*, this number is higher than expected, but cannot provide any further explanation.

6. parasite density need to be mentioned at-least these cases which were missed by qPCR

Answer: Microscopy data was obtained from health center microscopists and no parasite density data was provided (microscopists in Ethiopia are typically not WHO certified).

7. Author may provide the AUC for better understanding the test performance.

Answer: The AUC primarily used to describe the sensitivity and specificity of diagnostic tests. The aim of our study was not to determine the test performance of microscopy, and would be underpowered to do so. We this believe that including the AUC is not justified.

8. English language need to be taken care as line no. 241 -242 (Molecular screening for has identified a gap in the sensitivity for diagnosis of clinical P. falciparum cases and a substantial asymptomatic P. vivax reservoir) there is no meaning.

Answer: Thank you for spotting this typo; we have deleted ‘for’ and the sentence now reads (lines 272-273):

“Molecular screening has identified a gap in the sensitivity for diagnosis of clinical *P. falciparum* cases and a substantial asymptomatic *P. vivax* reservoir.”

9. Author need to provide the possible couse of fever as only 5% clinical cases were found positive for malaria parasite.

Answer: Many pathogens can cause febrile illness, and we did not test for any other infections than *P. falciparum* and *P. vivax*. We thus cannot provide possible causes of non-malarial fevers.

10. Author concluded that Prevention of onward transmission from the asymptomatic P. vivax reservoir might be achieved through improved vector control, however the current manuscript doesn't have objective to asses the vector control measures. Therefore, it can be discussed in the discussion section not to be in conclusion section.

Answer: We agree with the reviewer that this statement was too specific and have changed it as follows (lines 275-276):

*“P. vivax* control also needs to focus on prevention of onward transmission from the asymptomatic reservoir.”
