# Supplementary material for: qPCR in a suitcase for rapid Plasmodium falciparum and Plasmodium vivax surveillance in Ethiopia
Source: PLOS Glob Public Health. 2022 Jul 27;2(7):e0000454. doi: 10.1371/journal.pgph.0000454 (PMC10021179; doi:10.1371/journal.pgph.0000454)
Supplement: Attachment — Submitted filename: PLOS_Revision2_ResponseToReviewers.docx [file pgph.0000454.s005.docx]

We thank the reviewers for the additional comments. We are confident we were able to address all of them.

Journal/Reviewer comments are in black.

Our responses are in blue.

1. We have noticed that you have a list of Supporting Information legends (Supplementary File S1: Laboratory protocols) in your manuscript. However, there are no corresponding files uploaded to the submission. Please upload them as separate files with the item type 'Supporting Information'.

We apologize for not uploading the file correctly. We have now uploaded it.

2. We have noticed that you have uploaded Supporting Information files (Koepfli_PLOSGPH_Questionnaire.docx), but you have not included a list of legends. Please add a full list of legends for your Supporting Information files after the references list.

We have included the following caption:

“Supplementary File S3: PLOS’ questionnaire on inclusivity in global research”

3. Please review your reference list to ensure that it is complete and correct. If you have cited papers that have been retracted, please include the rationale for doing so in the manuscript text, or remove these references and replace them with relevant current references. Any changes to the reference list should be mentioned in the rebuttal letter that accompanies your revised manuscript. If you need to cite a retracted article, indicate the article’s retracted status in the References list and also include a citation and full reference for the retraction notice.

We are not aware that we have cited any papers that were retracted. We have cited a few webpages, maybe those created an alert during the reference check.

Reviewer #1: Reviewer's comments are well-addressed by author.

Thank you for your feedback!

Reviewer #2: 1. Author advocating the qPCR technology and still putting the methods as supplementary file, I believe it should be in main file.

As mentioned above, the file was not uploaded by mistake. The supplementary file contains a detailed step-by-step protocol. We believe this to be more useful than a plain text if included in the methods section of the manuscript. Researchers can print out the supplementary file and get started right away.

2.It is evaluation of the techniques so the parasite density must be required which is not done during the study, therefore, I would recommend that this study may consider as pilot study and validation study with comprehensive methodology covering all the aspect need to be done in future. According title of the manuscript may be modified.

As explained before, obtaining valid estimates of parasite density requires additional experiments and running external controls. Unfortunately, many published studies fail to do these experiments, and as a result, parasite densities reported by different labs can never be compared. The main questions of the current study were the number of clinical infections missed by routine diagnosis, and the prevalence of patent and subpatent asymptomatic infections. While parasite density affects the ability to detect them by microscopy or RDT, density per se was not a research question of the current study. For these reasons, we prefer not to report parasite densities rather than densities that are likely incorrect.

For the main questions outlined above, all details and methodology are comprehensively reported. It is clear that this is a research article and not a policy brief. We thus believe the title as stated represents the study correctly.
